# Lack of Evidence for Transmission of *Verticillium dahliae* by the Olive Bark Beetle *Phloeotribus scarabaeoides* in Olive Trees

**DOI:** 10.3390/pathogens10050534

**Published:** 2021-04-29

**Authors:** Ibrahim ElDesouki-Arafat, Hani K. Aldebis-Albunnai, Enrique Vargas-Osuna, Antonio Trapero, Francisco J. López-Escudero

**Affiliations:** 1 Horticulture Research Institute (HRI), Agricultural Research Center (ARC), Giza 12619, Egypt; z82elari80@gmail.com; 2 Departamento de Agronomía ETSIAM, Campus de Rabanales, Universidad de Córdoba, Edificio C4, 14071 Córdoba, Spain; cr2alalh@uco.es (H.K.A.-A.); cr1vaose@uco.es (E.V.-O.); ag1trcaa@uco.es (A.T.)

**Keywords:** fungus–insect interaction, *Olea europaea*, Scolytidae, Verticillium wilt

## Abstract

Verticillium wilt of olive, caused by *Verticillium dahliae* Kleb., is one of the most important diseases affecting olive crops in the Mediterranean area. With the aim to evaluate the role of *Phloeotribus scarabaeoides* (Bernard) (olive bark beetle) as a dispersal vector of *V. dahliae*, several experiments were conducted in semi-controlled conditions from May 2009 to April 2012. Groups of olive trees (2.5-year-old) certified free from *V. dahliae* were covered by a mosquito net and exposed to adults of *P. scarabaeoides* by three different ways: (1) branches or trunks collected in several olive orchards from trees severely affected by Verticillium wilt and showing apparent entry holes (mating galleries) of *P. scarabaeoides*; (2) adults of olive bark beetle extracted from damaged branches collected in the field; (3) adults from damaged branches that were superficially inoculated with *V. dahliae*. The fungus *V. dahliae* was not detected either by microbiological and molecular techniques from shoots of olive trees with galleries of the insect or from any of the tissues of the collected beetle adults from the galleries. Additionally, Verticillium wilt disease symptoms were not observed in olive trees exposed to the olive bark beetles. Moreover, the pathogen was never detected from any beetle adults that were recovered from the mating galleries of branches or trunks collected in several olive orchards from trees severely affected by Verticillium wilt. We conclude that *P. scarabaeoides* is not a vector of *V. dahliae* under the investigated experimental conditions.

## 1. Introduction

Verticillium wilt of olive (VWO), caused by *Verticillium dahliae* Kleb., is one of the major constraints for olive growing throughout the Mediterranean basin [1,2]. Particularly, the disease is severe in the Guadalquivir Valley in southern Spain (Andalusia), the world’s leading area for the production of olive oil and table olives (more than 1.5 million ha of olive orchards). In this area, the pathogen causes severe economic losses and kills thousands of trees annually. Thus, recent surveys on Verticillium wilt affected orchards in this region revealed mean disease incidence of 12%, 22%, and 24% in the three main olive producing provinces, Sevilla, Córdoba, and Jaén, respectively [3].

Some of the major causes of the VWO prevalence are the use of infested soils due to the previous crop of susceptible hosts of the pathogen and/or the infestation of healthy soils with inoculum coming from several outside inoculum sources. There were reported a number of means of dispersal of the pathogen in olive growing areas, mainly related to the movement of infective and survival structures (microsclerotia) that *V. dahliae* releases into the soil at the end of the disease cycle. Therefore, microsclerotia can be spread within or between olive orchards by the movement of infested soil or infected plant debris due to wind and rainfall or by the use of contaminated machinery [4,5,6,7,8]. In addition, dispersal by cultural practices, such as the use of infested animal manure as amendments in olive plantations [9], leaves and dried inflorescences from wilted trees [10], and irrigation water [11,12] were demonstrated. Besides these dispersal means, also the use of infected planting material is considered as a major factor in pathogen dispersal [13].

In soil, microsclerotia germinate and infect the roots of olive trees, initiating tissue colonization that leads the pathogen to the xylem vessels. There, the fungus produces mycelia and conidia that colonize the xylem. The infection process is favored by the moderate temperatures (20–25 °C) present in the conditions of southern Spain during spring and autumn months. If the density of the *V. dahliae* in the soil and its virulence are high, the olive cultivar presents high susceptibility to the fungus, and the favorable period for the development of infections is long; an extensive and consistent colonization of the vascular tissues is achieved during these weeks. Consequently, the tracheomycosis of the plant occurs, which interrupts water transport, subsequently causing defoliating, chlorosis, wilting of stems and branches of the tree, and, eventually, tree death [14,15].

*Phloeotribus scarabaeoides* (Bernard), the olive bark beetle, is a small xylophagous insect (Coleoptera: Scolytidae) widely spread in the Mediterranean basin and considered a secondary pest in olive groves [16]. The olive bark beetle attacks young olive branches in diseased, damaged, or weakened trees still attached to the stem or detached pruned branches as debris that are left in the field or collected and piled by farmers for fireplaces. In southern Spain, wintering adults have their refugee in early spring and look for olive branch debris from tree pruning or from declining olive trees. They perforate the bark and excavate a place for mating, which consists of a number of sub-cortical galleries where females deposit the eggs (mating galleries). After an incubation period from 8 to 13 days, hatched larvae excavate new galleries that are perpendicular to the maternal gallery. From April to the middle of May, depending on the weather, new adults come out from mating galleries, causing numerous and recognizable exit holes. These adults attack new plants producing feeding galleries, easily recognizable by the sawdust exuded from galleries and deposited around entrance holes. They remain in the feeding galleries after the end of summer (end of September or beginning of October), then adults leave them and excavate new small holes in olive wood or in the axils of the vegetative buds in which they overwinter, originating the attack the following spring [16].

Several plant pathogenic fungal species can be efficiently transmitted by xylophagous insects [17]. Nevertheless, only few cases are directly associated with vascular diseases. The most representative example of a vascular disease caused by a beetle-transmitted fungus is Dutch elm disease caused by *Ophiostoma ulmi* (Buisman) Nannf., which is transmitted by *Scolytus* spp. [18]. Adults of *Scolytus* spp. transport conidia of the pathogen stuck to their bodies when they leave the mating galleries drilled in diseased elm trees and go to healthy trees to make feeding galleries. This fungus–insect association is not a simple transmission but a mutualistic relationship between the beetle and the pathogen, which does not appear to occur in the case of *V. dahliae*.

In the case of *Verticillium* spp., there is only little information about its transmission by insects [19,20,21,22,23,24]. One of the examples is alfalfa, in which certain insects, such as pea aphid, alfalfa weevil, migratory grasshopper, two-striped grasshopper, and alfalfa leafcutting bee, can serve as vectors for effective transmission of the relative pathogen *V. albo-atrum* [2]. Interestingly, [19,21] demonstrated the transmission of *V. dahliae* by xylophagous insects *Scolytus intricatus* (Ratz.) and *Xyleborus dispar* (F.) when studying the causes of oak decline in Italy. Oak, however, is a rare host of *V. dahliae*. Moreover, recent studies conducted by [24] demonstrate that *Eucryptorrhynchus brandti* Harold (Coleoptera: Curculionidae) has the ability to carry and transfer *V. nonalfalfae* to *Ailanthus altissima* (tree of heaven) in a laboratory setting, initiating plant seedling infection after external contact with the fungus.

In this context, the vascular nature of infections of *V. dahliae* in olive and the life cycle of *P. scarabaeoides* could suggest an interaction between the two organisms regarding the spread of *V. dahliae* inoculum from Verticillium wilt affected olive trees to healthy trees. This hypothesis was supported by previous observations in fields doubly damaged by the olive bark beetle and VWO in several locations in Andalusia (southern Spain).

Therefore, the objective of the present study was to assess the possible role of *P. scarabaeoides* as a vector for transmission of *V. dahliae* in olive orchards. If it is so, it would explain why it is common to find old olive orchards (more than 60 years old) affected by VWO, when these orchards were established in pathogen free soil and apparently out of the influence of external contribution of *V. dahliae* inoculum by anyone of the dispersal means indicated above.

## 2. Results

### 2.1. Presence of V. dahliae in Olive Branches or Olive Bark Beetles from Olive Orchards (Experiment I)

*V. dahliae* was isolated from 20% of branches of olive trees showing severe symptoms of VWO and apparent entry holes of mating galleries produced by the olive bark beetle, which were collected from the four olive orchards surveyed during April and May 2009.

The pathogen was not isolated from the external surface of the integument tissues of any of the 50 adult beetles recovered from the mating galleries of these branches after plating on malt agar culture media with antibiotics. Similarly, *V. dahliae* was not present in the inner tissues of another 50 beetles tested. Molecular detection of the pathogen in adult of beetles did not yield positive results in any cases.

### 2.2. Inoculation of Olive Trees by Adults of the Olive Bark Beetle (Experiment II)

Experiment II, conducted from May 2009 to March 2010, did not yield positive evidence about the possible transmission of *V. dahliae* from olive branches affected by VWO to healthy olive trees by means of the olive bark beetle. Therefore, beetle adults from olive branches (T1) or artificially inoculated with *V. dahliae* (T2) produced feeding galleries in healthy olive trees, but these trees were not infected by *V. dahliae*.

The number of feeding galleries per tree produced *by P. scarabaeoides* in each block of each treatment during June 2009 is shown in Table 1. The average number of feeding galleries was significantly higher (*p* < 0.0001) in trees of T1 than in T2, with 164 and 42 galleries, respectively. In T2, 70% of the released adults produced feeding galleries in the healthy trees.

The number of holes per shoot produced by *P. scarabaeoides* in each tree was recorded during a monthly sampling conducted from September 2009 to March 2010, aimed to extract adults and to detect the presence of the pathogen superficially or within these insects (Table 2). Average number of entry holes per shoot was significantly higher (*p* < 0.0001) in trees of the treatment T1 (7.7) than in T2 (3.5). The two control trees showed an average of 16.7 entry holes.

During the period from September 2009 to March 2010, no symptoms of Verticillium wilt were observed in any olive trees, whether exposed to olive bark beetles exposed to Verticillium wilt and putative vectors or in control trees. Moreover, *V. dahliae* was not detected using microbiological and molecular techniques for isolation in any of the studied samples collected from the healthy olive trees and nor in adults of olive bark beetle extracted from feeding galleries produced in shoots; also, the pathogen was not isolated from control.

### 2.3. Inoculation of Olive Trees by Adults of the Olive Bark Beetle (Experiment III)

In Treatment T3, the first inoculation of trees with *V. dahliae* by stem injection did not produce consistent symptoms of VWO during summer 2010. Furthermore, the pathogen was not detected in the sampled shoots from inoculated trees in May 2010, likely due to its inability to extensively colonize plant tissues. On the contrary, when the inoculation was repeated in October 2010, 13 of the 20 inoculated olive trees showed typical VWO symptoms 3 weeks after inoculation. Isolation on PDA plates from affected tissues confirmed the presence of the pathogen in 4 out of the 13 symptomatic trees.

Shoots of the six healthy trees introduced in the net of T3 were perforated by adults of the olive bark beetle coming from the inoculated trees during October 2010 to June 2011. Similarly, the four healthy trees introduced in the net of the T4 were perforated by adults from their respective trees that were artificially inoculated with *V. dahliae* during the same period. Finally, the eight healthy trees included in the net of T5 were severely damaged by the adults of *P. scarabaeoides* coming from olive branches infected by Verticillium wilt and collected from an olive orchard in the municipality of Almódovar. The total number of these galleries was recorded at the end of June 2011 (Table 3). Results showed significant differences (*p* < 0.05) between treatments that ranged from 50.0 (T5) to 32.8 (T4) feeding or mating galleries.

None of the healthy trees introduced in the net of treatments T3, T4, and T5 exhibited wilt symptoms over the entire period of observation of the Experiment III (October 2010 to March 2012).

Samplings of affected shoots from the healthy trees of the three treatments were conducted in November 2011 and March 2012. The average number of holes (feeding and mating galleries) per shoot showed significant differences (*p* < 0.0001) between treatments, reaching 5.5, 3.4, and 8.2 holes, respectively (Table 4). *V. dahliae* was not detected by isolations in PDA or PCR technique in any of the studied samples collected from the initial healthy olive trees, demonstrating that plants were not infected. Similarly, the pathogen could not be isolated from inner or superficial tissues of any of the olive bark beetles recovered from galleries in collected shoots.

## 3. Discussion

This is the first study evaluating the role of *P. scarabaeoides*, the olive bark beetle, as a potential vector of *V. dahliae* in olive orchards. The initial hypothesis was based on the activities related to the biological cycle of this beetle (Coleoptera, Scolytidae) and the cycle of infection of *V. dahliae*. The main means of dispersal of *V. dahliae* take place through the movement of its survival structures, called microsclerotia, during the phase of the cycle in which the pathogen is not in contact with the plant. Nevertheless, once the microsclerotia infect the root of the plant, the pathogen causes the infection of the vascular tissues through the production of mycelium and conidia that progressively colonize the xylem, where it remains viable. It constitutes the basis of the initial hypothesis of this work according to which the adults of the olive bark beetles could acquire and later transmit the pathogen to other healthy plants during the biological coincidence of both (production of feeding or mating galleries of *P. scarabaeoides* close or in the invaded xylem vessels by *V. dahliae*) [16]. We were unable to find any evidence that pointed to olive bark beetles as vectors of *V. dahliae* following sampling of adult beetles from infected olive trees in several olive orchards affected by the two agents: the fungal pathogen and the olive bark beetle. This was despite samples of shoots and branches colonized by the beetles confirmed as having high disease incidence. The validity of this negative result was supported by the fact that sampling was conducted at the most favorable time (April and May) for disease development under our conditions [25].

Despite this outcome, experiments were conducted over a long period (March 2009 to May 2012) in order to explore in several years all the possibilities of inoculation by the insect when its biological cycle coincided with the most favorable period for infection and VWO symptom development. These periods were mainly spring, early summer, and fall.

The experimental design, using mosquito nets, allowed the isolation of the treatments, where the movement of adult olive bark beetles between healthy and artificially wilted olive trees was guaranteed. Indeed, the quantification of entry holes caused by the insect in the shoots of the healthy trees included in the nets was a main parameter for demonstrating that the inoculation of *V. dahliae* might have occurred if the insect was able to transmit the pathogen (Table 1, Table 2, Table 3 and Table 4). Moreover, the role of the olive bark beetle as a vector of *V. dahliae* was assessed by evaluating disease symptoms of VWO and the presence of the pathogen in tissues of olive trees invaded by adult insects. Finally, adult beetles were also extracted from mating and feeding galleries excavated in shoots of olive trees artificially or naturally infected by *V. dahliae* that showed severe VWO symptoms. Thereafter, the presence of the pathogen in superficial and internal tissues of these beetles was investigated.

The presence of feeding galleries in the shoots sampled during the period from September 2009 to March 2010 (Experiment II) shows that the insect was active and could move from one tree to another. However, the absence of disease symptoms and infections of trees or contaminated beetle adults showed that the inoculation did not occur.

A third experiment was performed in which the olive trees used in treatments T3 and T4 were inoculated twice by stem injection with *V. dahliae*. The aim was to uniformly infect the tissues in which *P. scarabaeoides* was actively producing mating and feeding galleries and, thereafter, introduce in the nets new, healthy olive trees. The first inoculation did not produce consistent symptoms of VWO. This was likely due to high temperatures registered at the beginning of summer, which impeded an extensive colonization of tree tissues by the pathogen. Furthermore, the stem injection inoculation of olive plants generally results in colonization not as intense as when using root inoculation in controlled conditions [26,27]. Nevertheless, the second inoculation, conducted in October 2010, caused typical VWO symptoms in the olive trees, but the isolation on PDA plates from affected tissues confirmed the presence of the pathogen in only 4 out of the 13 symptomatic trees. This slow rate of isolations is sometimes common after artificial inoculations of woody host of *V. dahliae* such as olive tree, since its slow growth makes it tough to isolate if any other fungi are present.

In this experiment, the number of feeding and mating galleries caused by beetles (Table 3 and Table 4) showed a similar trend in Experiment II regardless of the releasing insects. Although the number of feeding galleries was variable in the three treatments, we think that this number was high enough to transmit the pathogen if the olive bark beetle was an effective vector of the pathogen.

Healthy olive trees exposed to olive bark beetles in the described experimental conditions showed Verticillium wilt disease symptoms. Similarly, the pathogen could not be isolated from any of the tissues of sampled shoots where the insects made galleries or from any of the tissues of the beetle adults collected from the galleries.

Ascomycetous fungi associated with bark beetles include the genera *Ophiostoma*, *Ceratocystis*, and *Ceratocystiopsis* and the anamorphic genera *Graphium* and *Leptographium* [28,29,30]. Several fungal species causing different diseases in tree hosts can be efficiently transmitted by xylophagous insects [17]. Nevertheless, only a few cases are directly associated with vascular diseases. The most representative example of a vascular disease caused by a beetle-transmitted fungus is Dutch elm disease caused by *Ophiostoma ulmi*, which is transmitted by *Scolytus* spp. [18]. Diseases caused by *Ceratocystis* include vascular wilts, *Ceratocystis fimbriata*, Ellis & Halst., one of the most virulent and economically important vascular pathogens in many agricultural crops and forest trees [31], which is frequently associated with insects on *Populus* [32] and *Prunus* [33].

We did not find scientific evidence in literature that expressly indicates that Verticillium dahliae is capable of surviving in the adults or the larval stages of the olive bark beetle. In fact, the starting hypothesis did not consider such survival per se but considered that the adults of the insect could constitute a support for the transfer of the conidia from one tree to another. The hypothesis was based on a possible parallelism with the mutualistic association that occurs in the pathosystem Ophiostoma novo ulmi/Bark beetles of *Scolytus* spp. Geoffroy, *S. scolytus* (F.) and *S. multistriatus* (Marsham), the main vectors of the pathogen which causes the Dutch elm disease.

Adaptations for insect dispersal (ascomata and conidiomata with sticky spore drops) arose frequently in the evolution of Ascomycetes [34]. In this case, O. novo-ulmi presents during its life cycle two asexual forms that produce asexual spores (conidia). First, in infected trees (dying or recently dead), the mycelium of the fungus produces sticky conidia (Graphium-type spores) in the galleries created by beetles just under the bark. These spores are produced at the tips of synnemata (coremia), dark brown to black stalks about 1–2 mm in length, each composed of aggregated hyphae [35,36,37].

When the new beetles emerge as adults from infected elms, they acquire the conidia of the fungus on and in their bodies and carry them to new elm trees, where they produce feeding tunnels in direct contact with the xylem vessels of the host. The pathogen invades the vascular tissues where they germinate to produce mycelium and cephalosporium-type spores (yeast phase) that move and colonize the vessels, causing tree wilt [38,39]. In the case of *V. dahliae*, there is very little information about its transmission by insects. For example, the transmission of *Verticillium dahliae* by xylophagous insect vectors *Scolytus intricatus* (Ratz.) and *Xyleborus dispar* (F.) was demonstrated when studying the causes of oak decline in Italy [19,21]. Oak tree, however, is a rare host of *V. dahliae*. Nevertheless, *V. dahliae* does not produces similar asexual structures such as the mentioned synnemata produced by *Ophiostoma* spp that would cause the pathogen to contact the adult beetle bodies when they abandon the galleries looking for a new, healthy tree. The pathogen enters their host plants through the roots, and, following penetration, the fungi colonize the cortical cells from where hyphae migrate intercellularly toward the vascular parenchyma cells and invade the xylem vessels, where Yadeta and Thomma (2013) are mostly restricted. Therefore, the conidial production is limited to the vascular vessels, and the opportunity of beetle adults to get in contact with and to acquire conidia of the pathogen is restricted to this moment.

We can conclude that *P. scarabaeoides*, the olive bark beetle, is not a vector of *V. dahliae*, the pathogen causing Verticillium wilt of olive, in the investigated experimental conditions. The causes are probably related to the inefficiency of the beetle to acquire inoculum from diseased trees (attached to its body or inside its digestive tract) and/or transport and deposit viable pathogen conidia in the appropriate site, close to or inside xylem vessels, which would allow pathogens to establish the infection.

## 4. Materials and Methods

During the period May 2009 to April 2012, three experiments were conducted in commercial olive orchards and in semi-controlled conditions in a net-house at the Campus of Rabanales, University of Córdoba.

### 4.1. Experiment I

**Figure 1:**
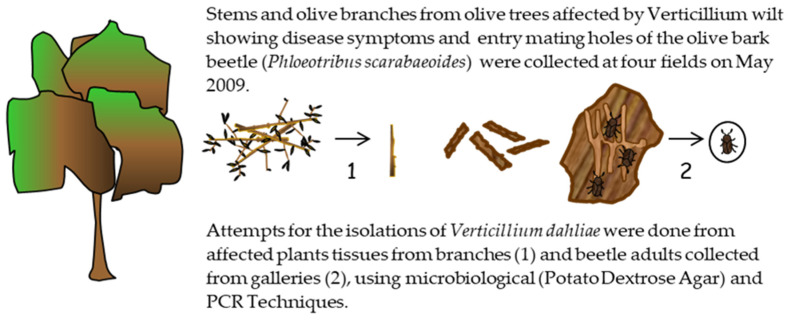
Sampling and isolation of *Verticillium dahliae* from stems of olive trees affected by Verticillium wilt of olive and attack by *Phloeotribus scarabaeoides* at Experiment I (May 2009).

A field survey was conducted to collect olive branches showing wilt symptoms and/or entry holes (mating galleries) of *P. scarabaeoides* in four olive orchards severely affected by VWO, located in the provinces of Córdoba (municipalities of Almodóvar, Montilla and Santaella) and Jaén (municipality of Andújar) (Figure 1). The tree samples were used to check for the presence of the pathogen on the potential vector, and as a source of beetles for Experiments II and III. In addition, 3 to 6 shoots or branches (between 8 and 15 cm in diameter) per olive tree were collected from 12 olive trees from each orchard at two sampling dates during period April and May 2009.The presence of *V. dahliae* in sampled branches was assessed by microbiological isolations. For these analyses, samples of affected woody tissue were washed in running tap water for 20 min, bark was removed, and tissue was surface disinfected in 0.5% sodium hypochlorite for 40 s. Wood chips were placed on potato dextrose agar (PDA) plates and incubated at 24 °C in the dark for 5 to 6 days. In addition, the detection of the pathogen in the samples was tested by molecular analyses based on nested-PCR [34].

For collecting adults of *P. scarabaeoides* from the plant samples, branches were stored in big plastic boxes (38 cm length, 30 cm width, and 14 cm high) at room temperature in the laboratory. After emergence, adults were collected in small plastic boxes, and microbiological isolations of *V. dahliae* from inner and superficial tissues of them were done using two different methods.

(1) To check for the superficial presence of the fungal pathogen on the insect, 50 adults were chosen at random from those that emerged in the plastic boxes containing plant material from the different surveyed fields and placed onto plates (five adults per plate) of Malt Agar culture media amended with 1 mL of tetracycline (Sigma Aldrich) (0.05 g/mL), 1 mL of streptomycin (Sigma Aldrich) (0.60 g/mL), 5 mL of Ciclohexamida PB (Panreac) (0.05 g/mL), and 1 mL of dodine pestanal (Sigma Aldrich) (0.1 g/mL) [35,36].

(2) Another 50 adults were surface disinfected in 1.5% sodium hypochlorite for 40 s and then dried on sterile filter paper. Then, they were separately immersed in 5 mL of sterile distilled water and individually ground with a sterile mortar and pestle. The resulting mass per insect was diluted (0.1, 0.01, and 0.001), and 300 µL of each suspension was spread onto culture plates of the same medium. Plates were incubated at 22–24 °C for 7 days. Finally, the detection of the pathogen in the ground samples was also attempted using the nested-PCR technique [34].

### 4.2. Experiment II

**Figure 2:**
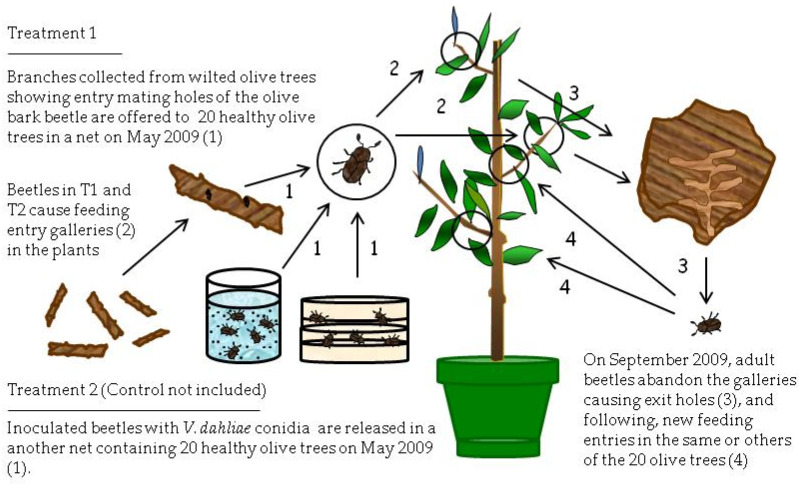
Process of inoculation with *Verticillium dahliae* of healthy olive trees using natural and artificially inoculated adult beetles of *Phloeotribus scarabaeoides* (olive bark beetle) in Treatments T1 and T2 at Experiment II (May 2009–March 2010).

This experiment was carried out from May 2009 to March 2010 (Figure 2). Healthy, 2.5-year-old olive trees (1.6–2.0 m height) of the Spanish cultivar Picual, susceptible to Verticillium wilt [26], were used. These trees were acquired from a commercial nursery in Villanueva del Duque (north of Córdoba province, Spain). Nursery trees were propagated by rooting under mist of soft-wood cuttings obtained from certified disease-free olive mother trees. Trees grew in plastic pots (20 L in volume) in a lathhouse.

The experiment consisted of the following treatments. Treatment 1 (T1) was composed of four groups of four VWO-free olive trees. Each group of four trees was individually covered by a carbon fiber mosquito net and exposed early in May to seven branches affected by VWO that showed entry holes (mating galleries) by the bark beetle (Figure 2). The hypothesis was that adults, developed into mating galleries (supposedly infested by conidia of *V. dahlia*) after exiting the mating galleries from May to June, would produce entry holes (feeding galleries) in branches or main trunk and inoculate the healthy trees with *V. dahliae*.

Treatment two (T2) also consisted of four groups of four similar olive trees covered by mosquito nets, but, in this case, trees were exposed to bark beetle adults previously infested by the pathogen that were released inside the nets (Figure 2). The isolate of *V. dahliae* coded as V117, a highly virulent cotton defoliating strain from the collection of the Plant Pathology Laboratory of the Agronomy Department at the University of Córdoba [37], was used to inoculate the bark beetle adults. The pathogen was transferred from PDA slants, where it was maintained at 4 °C, to PDA plates by spreading the mycelium uniformly over the entire surface of each plate. Plates were incubated at 25 °C for 6 days in the dark. Infestation of beetles was achieved by confining adults for 7 h in a sandwich composed of two PDA plates with active cultures of *V. dahliae* facing each other and sealed with Parafilm^®^ (American National Can). In the evening, one plastic cap containing 60 olive bark beetles from the PDA plates was placed on the soil near each tree. This method was successfully used in previous works [23] that demonstrated that adults of fungus gnats and moth flies externally acquired the conidia of *V. dahliae* after exposure to the cultures and subsequently became efficient distributors of the pathogen.

Finally, the control treatment consisted of two groups of two healthy olive trees. The first group (Control 1) was covered by an individual mosquito net and exposed to five *V. dahliae*-free branches that showed entry holes (mating galleries) of the olive bark beetle. A second group (Control 2) remained uncovered and not exposed to *P. scarabaeoides* adults or damaged olive branches by the beetles, and it was used for confirming that plants were free from infections of *V. dahliae*.

Several days after inoculation, the number of entry holes (feeding galleries) produced by beetles was recorded in the healthy trees used for the different treatments. During the following months after beetle inoculations (July to September 2009), trees were periodically inspected to identify Verticillium wilt disease symptoms such as wilt, chlorosis, leaf and shoot necrosis, or defoliation.

In September 2009, the damaged branches that were used as a beetle source for healthy trees were removed. By this time, beetles were exiting the galleries they made on the healthy trees, on which they fed during summer, to look for new trees in which to make feeding galleries. Removing branches forced adults to make new feeding galleries in the same trees.

From September 2009 to March 2010, three shoots per tree were collected, and the number of feeding galleries was recorded. The sampling was only conducted in trees with high numbers of entry holes, rejecting trees slightly damaged. Thus, totals of 6, 42, and 24 shoots were collected from 2, 14, and 8 trees from Control 1, T1, and T2, respectively. Adult beetles were periodically extracted from the feeding galleries of these plants to check if the insects were superficially or internally infested by *V. dahliae* and/or if olive shoots were infected by the inoculum presumably transmitted by the beetles. Therefore, molecular analyses and microbiological isolations from plant tissues and from adults were done using the nested-PCR technique [34].

### 4.3. Experiment III

**Figure 3:**
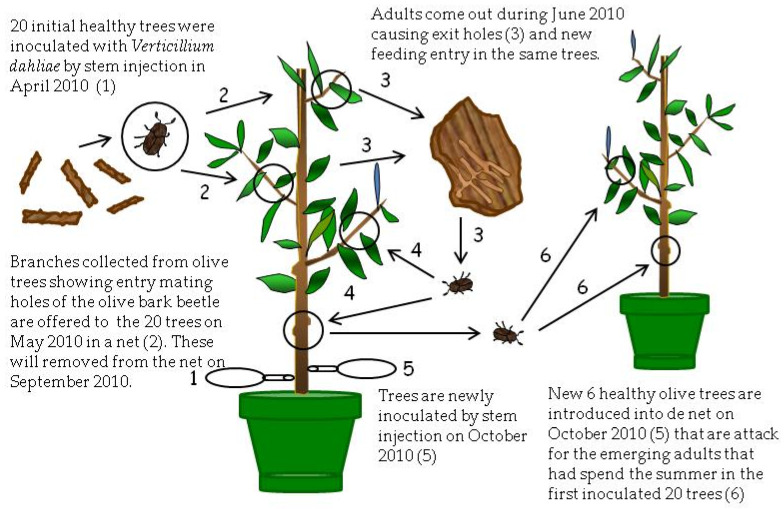
Process of inoculation with *Verticillium dahliae* of healthy olive trees using stem injection and attack by adult beetles of *Phloeotribus scarabaeoides* (olive bark beetle) in Treatment III at Experiment III (April 2010–March 2012).

**Figure 4:**
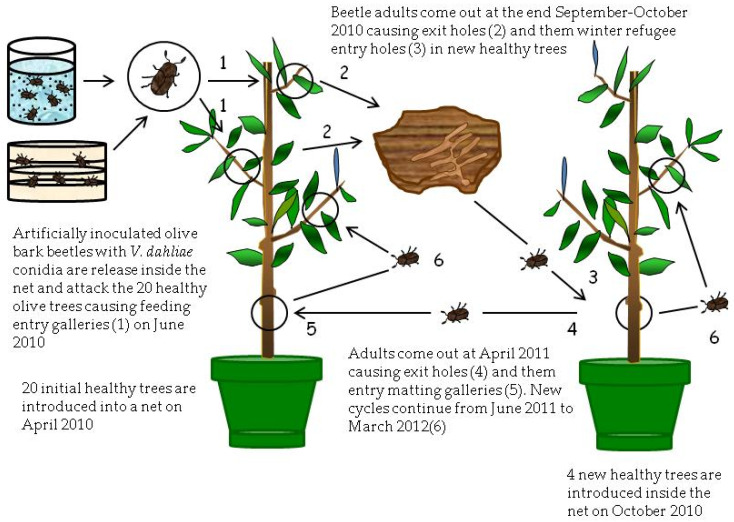
Process of inoculation with *Verticillium dahliae* of healthy olive trees using artificially inoculated adult beetles of *Phloeotribus scarabaeoides* (olive bark beetle) in Treatment IV at Experiment III (April 2010–March 2012).

This experiment was carried out from April 2010 to March 2012 in which olive trees from Experiment II were used because Verticillium was not detected in either plants or beetles. The experiment consisted of three treatments (Figure 3 and Figure 4).

In the first treatment (T3), twenty trees chosen at random from the treatments T1 and T2 of the Experiment II were placed in April (2010) inside a new mosquito net and were inoculated by stem injection with 50 mL of a conidial suspension of 10^5^ conidia/mL of the V117 *V. dahliae* isolate [27] (Figure 3). To produce conidia of *V. dahliae*, 15 mL of sterilized distilled water was poured over active colonies of *V. dahliae* on the PDA plates and then the surface was gently rubbed with a sterilized glass rod. The mycelial and conidial suspension was passed through a double sterilized cheesecloth yielding a water-suspension of conidia that was finally adjusted to a concentration of 10^7^ conidia/mL. Four weeks after inoculation (May 2010), thirty *V. dahliae*-free branches collected from the Montilla field with entry holes (mating galleries) of the insect were again introduced in the mosquito net. Adults emerged at the beginning of June 2010 and infested olive trees again, producing feeding galleries (Figure 3). The aim of these inoculations was to increase pathogen colonization of tissues and the number of beetles in these trees. After inoculations (May 2010), sampling of plant tissue was conducted to confirm the presence of the pathogen in affected shoots by microbiological isolations in PDA. Moreover, during the following months, trees were periodically inspected to assess VWO symptoms and damages caused by the insect. In September 2010, introduced branches on May 2010 were removed from inside the mosquito net. In October 2010, trees of this treatment were again inoculated with *V. dahliae* by trunk injection, similar to the previous April. Afterwards, six new, healthy trees with the same characteristics of those used initially in Experiment II and acquired from the same commercial nursery were introduced inside the net. Again, the hypothesis was that adults that spent the summer within inoculated trees and were now abandoning the feeding galleries would be able to transmit the pathogen to the new, healthy trees they were using to build new feeding galleries (Figure 3). In November 2010, microbiological isolations on PDA plates of symptomatic tissues were conducted using trees of this treatment to confirm the presence of the pathogen in symptomatic shoots.

The second treatment of this experiment (T4) also consisted of another group of twenty trees, chosen at random from treatments T1 and T2 of the Experiment II that were introduced in April 2010 in a new mosquito net (Figure 4). Trees were exposed during June 2010 to 420 adults of *P. scarabaeoides*. These adults were extracted from damaged olive branches collected in the Montilla field and infested with *V. dahliae* by two different ways. Half of the adults were infested as described for Experiment II using a sandwich of *V. dahliae* PDA culture plates. The other half was infested by immersion for 40 s in a conidial suspension of 10^8^ conidia/mL of the V117 isolate. Infested beetles were released into the mosquito net to produce new entry holes (feeding galleries) in the twenty olive trees during the summer feeding. Finally, during October 2010, four healthy trees were introduced inside the net of the treatment T4 to expose them to attack by the beetles from the initial twenty olive trees (Figure 4).

A third treatment (T5) (not illustrated by a figure) consisted of eight healthy trees that were exposed to thirty olive branches damaged by *P. scarabaeoides* that showed clear VWO symptoms in April 2011. These branches were collected from an olive orchard in the municipality of Almodóvar near Cordoba.

The control treatment in this experiment consisted of four healthy trees not exposed to the bark beetle.

In June and July 2011, the new generation of beetles came out from trees inside the corresponding net, and the number of entry holes (feeding and mating) produced in tree shoots by beetles was recorded. In addition, samplings of affected shoots from the healthy trees of the three treatments were conducted in November 2011 and March 2012 using the same methods explained above for Experiment II. In these samples, the number of holes (feeding and mating galleries) was recorded. Moreover, infections by *V. dahliae* of collected plant material were assessed using isolations on PDA and nested-PCR analyses. The same analyses were made for adults of the olive bark beetle recovered from galleries of collected shoots.

### 4.4. Statistical Analysis of Data

Analysis of variance (ANOVA) of evaluated parameters was performed using Statistix 9.0 program (Analytical Software, Tallahassee, FL, USA). Mean values were compared by the Fisher’s protected LSD test at *p* = 0.05. Prior to analysis, data were checked in regard to the three main assumptions (homogeneity of variances, normality, and distribution of residuals).

## Figures and Tables

**Table 1:** Number of feeding galleries per tree produced by *Phloeotribus scarabaeoides* in healthy olive trees in the Experiment II from May to June 2009.

Mosquito Net (n°)	Control 1	Treatment 1	Treatment 2
1	261.8	162.5	31.5
2	-	186.0	37.0
3	-	138.5	58.5
4	-	169.8	42.5
Mean	261.8	164.2 a	42.4 b

Healthy olive trees were arranged by groups of two plants (Control 1) or four plants (Treatments 1 and 2) inside mosquito nets, where trees were exposed to olive bark beetles. Control 1 = Olive trees invaded by olive bark beetles coming from non-wilted trees. Treatment 1 = Olive trees invaded by olive bark beetles coming from branches of olive trees affected by Verticillium wilt. Treatment 2 = Olive trees invaded by olive bark beetles superficially inoculated with *V. dahliae*. Mean values followed by the same letter were not significantly different according to the LSD test (*p* = 0.05).

**Table 2:** Number of feeding galleries per shoot produced by *Phloeotribus scarabaeoides* in healthy olive trees in the Experiment II from September 2009 to March 2010.

Mosquito Net (n°)	Control 1	Treatment 1	Treatment 2
1	16.7	8.9	1.3
2	-	5.8	1.7
3	-	7.4	4.8
4	-	8.8	2.8
Mean	16.7	7.7 a	3.5 b

Healthy olive trees were arranged by groups of two plants (Control 1) or four plants (Treatments 1 and 2) inside mosquito nets, where trees were exposed to olive bark beetles. Control 1 = Olive trees invaded by olive bark beetles coming from non-wilted trees. Treatment 1 = Olive trees invaded by olive bark beetles coming from branches of olive trees affected by Verticillium wilt. Treatment 2 = Olive trees invaded by olive bark beetles superficially inoculated with *V. dahliae*. Mean values followed by the same letter were not significantly different according to the LSD test (*p* = 0.05).

**Table 3:** Number of feeding and mating galleries per tree produced by *Phloeotribus scarabaeoides* in healthy olive trees in the Experiment III from October 2010 to June 2011.

Tree (n°)	Treatment 3	Treatment 4	Treatment 5
1	45	26	45
2	40	34	52
3	47	35	56
4	37	36	60
5	43	-	44
6	37	-	42
7	-	-	61
8	-	-	40
Mean	41.5 b	32.8 c	50.0 a

Treatment 3 = 20 olive trees inoculated by stem injection with *V. dahliae* in a mosquito net, in which 30 branches affected by *P. scarabaeoides* were introduced. Thereafter, six healthy plants were introduced inside the net. Treatment 4 = 20 olive trees inoculated by stem injection with *V. dahliae* in a mosquito net, in which 420 adults of *P. scarabaeoides* artificially infested with *V. dahlia* were released. Thereafter, four healthy trees were introduced inside the net. Treatment 5 = 8 healthy olive trees in a net in which 30 olive branches collected from the field damaged by *P. scarabaeoides* and infected by *V. dahliae* were introduced. Values in row followed by the same letter were not significantly different according to the LSD test (*p* = 0.05).

**Table 4:** Number of feeding and mating galleries per shoot produced by *Phloeotribus scarabaeoides* in healthy olive trees in the Experiment III in November 2011 and March 2012.

Tree (n°)	Treatment 3	Treatment 4	Treatment 5
1	6.0	3.0	6.3
2	6.0	4.0	9.0
3	6.0	3.0	8.0
4	3.0	3.7	8.3
5	6.0	-	8.7
6	5.7	-	8.3
7	-	-	9.0
8	-	-	8.0
Mean	5.5 b	3.4 c	8.2 a

Treatment 3 = 20 olive trees inoculated by stem injection with *V. dahliae* in a mosquito net, in which 30 branches affected by *P. scarabaeoides* were introduced. Thereafter, six healthy plants were introduced inside the net. Treatment 4 = 20 olive trees inoculated by stem injection with *V. dahliae* in a mosquito net, in which 420 adults of *P. scarabaeoides* artificially infested with *V. dahliae* were released. Thereafter, four healthy trees were introduced inside the net. Treatment 5 = 8 healthy olive trees in a net in which 30 olive branches collected from the field damaged by *P. scarabaeoides* and infected by *V. dahliae* were introduced. Values in row followed by the same letter were not significantly different according to the LSD test (*p* = 0.05).

## Data Availability

Not applicable.

## References

[1] López-Escudero F.J., Mercado-Blanco J. (2011). Verticillium wilt of olive: A case study to implement an integrated strategy to control a soil-borne pathogen. Plant Soil.

[2] Mercado-Blanco J., López-Escudero F.J. (2012). Verticillium wilt of olive and its control: The heat is on. Plant Soil.

[3] López-Escudero F.J., Roca J.M., Mercado-Blanco J., Valverde-Corredor A., Blanco-López M.A. (2010). Verticillium wilt of olive in the Guadalquivir Valley (Southern Spain): Relations with some agronomical factors and spread of Verticillium dahliae. Phy-topathol. Medit..

[4] Wilhelm S., Taylor J. (1965). Control of Verticillium wilt of olive through natural recovery and resistance. Phytopathology.

[5] Tjamos E.C., Botseas D. (1987). Occurrence of Verticillium dahliae in leaves of Verticillium-Wilted olive trees. Can. J. Plant Pathol..

[6] Tjamos E.C., Tsougriani H. Formation of Verticillium dahliae microsclerotia in partially disintegrated leaves of Verticillium affected olive trees. Proceedings of the 5th International Verticillium Symposium.

[7] Al-Ahmad M.A., Mosli M.N. (1993). Verticillium wilt of olive in Syria. EPPO Bull..

[8] Serrhini M.N., Zeroual A. (1995). Verticillium wilt in Morocco. Olivae.

[9] Lopez-Escudero F.J., Blanco-Lopez M.A. (1999). First Report of Transmission of Verticillium dahliae by Infested Manure in Olive Orchards in Andalucia (Southern Spain). Plant Dis..

[10] Trapero C., Roca L.F., Alcántara E., López-Escudero F.J. (2011). Colonization of Olive Inflorescences by Verticillium dahliae and its Significance for Pathogen Spread. J. Phytopathol..

[11] Rodríguez-Jurado D., Bejarano-Alcázar J. (2007). Dispersión de Verticillium dahliae en el agua utilizada para el riego de olivares en Andalucía. Boletín de sanidad vegetal. Plagas.

[12] García-Cabello S., Pérez-Rodríguez M., Blanco-López M.A., López-Escudero F.J. (2012). Distribution of Verticillium dahliae through watering systems in widely irrigated olive growing areas in Andalucia (Southern Spain). Eur. J. Plant Pathol..

[13] Thanassoulopoulos C.C. (1993). Spread of verticillium wilt by nursery plants in olive groves in the Halkidiki area (Greece). EPPO Bull..

[14] Schnathorst W.C., Mace M.E., Bell A.A., Beckman C.H. (1981). Life cycle and epidemiology of Verticillium. Fungal Wilt Diseases of Plants 1981.

[15] Trapero A., Blanco M.A., Barranco D., Fernández-Escobar R., Rallo L. (2010). Diseases. Olive Growing 2010.

[16] Alvarado M., Civantos M., Durán J.M., Barranco D., Fernández-Escobar R., Rallo L. (2010). Pests. Olive Growing 2010.

[17] Webber J., Gibbs J. (1989). Insect Dissemination of Fungal Pathogens of Trees. Insect-Fungus Interactions.

[18] Webber J.F. (2004). Experimental studies on factors influencing the transmission of Dutch elm disease. Investigación agraria. Sist. Recur. For..

[19] Riziero T., Ragazzi A. (1998). Association between fungi and xylophagous insects of declining oaks in Italy. Redia.

[20] Pegg G.F., Brady B.L. (2002). Verticillium Wilts 2002.

[21] Riziero T., Ragazzi A., Marianelli L., Sabbatini P. (2002). Insects and fungi involved in oak decline in Italy. Bull. OILB SROP.

[22] Huang H. (2003). Verticillium wilt of alfalfa: Epidemiology and control strategies. Can. J. Plant Pathol..

[23] El-Hamalawi Z. (2008). Acquisition, retention and dispersal of soilborne plant pathogenic fungi by fungus gnats and moth flies. Ann. Appl. Biol..

[24] Snyder A.L., Salom S.M., Kok L.T., Griffin G.J., Davis D.D. (2012). Assessing Eucryptorrhynchus brandti (Coleoptera: Curculio-nidae) as a potential carrier for Verticillium nonalfalfae (Phyllachorales) from infected Alianthus altissima. Bio. Sci. Technol..

[25] Montes F., Páez J.I., Vega J.M., Duhart M.E. (1997). Épocas de aislamiento de Verticillium dahliae Kleb en olivar en la provincia de Sevilla. Bol. San. Veg. Plagas.

[26] López-Escudero F.J., Del Río C., Caballero J.M., Blanco-López M.A. (2004). Evaluation of olive cultivars for resistance to Verticil-lium dahliae. Eur. J. Plant Pathol..

[27] López-Escudero F.J., Del Río C., Caballero J.M., Blanco-López M.A. (2007). Response of olive cultivars to stem puncture inocu-lation with a defoliating pathotype of Verticillium dahliae. HortScience.

[28] Harrington T.C., Schowalter R.D., Filip G.M. (1993). Biology and taxonomy of fungi associated with bark beetles. Beetle-Pathogen Interactions in Conifer Forests 1993.

[29] Upadhyay H.P., Wingfield M.J., Seifert K.A., Webber J.F. (1993). Classication of the ophiostomatoid fungi. Ceratocystis and Ophiostoma: Taxonomy, Ecology and Pathogenicity 1993.

[30] Wingfield M.J., Harrington T.C., Solheim H. (1997). Two species in the Ceratocystis coerulescens complex from conifers in western North America. Can. J. Bot..

[31] Kile G.A., Wingfield M.J., Seifert K.A., Webber J.F. (1993). Plant diseases caused by species of *Ceratocystis sensu stricto* and *Chalara*. Ceratocystis and Ophiostoma: Taxonomy, Ecology and Pathogenicity.

[32] Hinds T.E. (1972). Insect Transmission of Ceratocystis Species Associated with Aspen Cankers. Phytopathology.

[33] Moller W.J., DeVay J.E. (1968). Carrot as a species-Selective isolation medium for Ceratocystis fimbriata. Phytopathology.

[34] Blackwell M. (1994). Minute mycological mysteries: The influence of arthropods on the lives of fungi. Mycology.

[35] Terry T.A. (1989). Diseases of Shade Trees (Revised Edition).

[36] Okada G., Seifert K.A., Takematsu A., Yamaoka Y., Miyazaki S., Tubaki K. (1998). A molecular phylogenetic reappraisal of the Graphium complex ased on 18S rDNA sequences. Can. J. Bot..

[37] Santini A., Faccoli M. (2015). Dutch elm disease and elm bark beetles: A century of association. iForest.

[38] Webber J.F., Brasier C.M., Anderson J.M., Rayner A.D.M., Walton D. (1984). The transmission of Dutch elm disease: A study of the process involved. Invertebrate-Microbial Interactions.

[39] Yadeta K., Thomma B. (2013). The xylem as battleground for plant hosts and vascular wilt pathogens. Front. Plant Sci..

